# Public Engagement and Government Responsiveness in the Communications About COVID-19 During the Early Epidemic Stage in China: Infodemiology Study on Social Media Data

**DOI:** 10.2196/18796

**Published:** 2020-05-26

**Authors:** Qiuyan Liao, Jiehu Yuan, Meihong Dong, Lin Yang, Richard Fielding, Wendy Wing Tak Lam

**Affiliations:** 1 School of Public Health The University of Hong Kong Hong Kong; 2 School of Nursing The Hong Kong Polytechnic University Hong Kong; 3 School of Nursing The University of Hong Kong Hong Kong

**Keywords:** risk communication, social media, epidemic, COVID-19, pandemic, outbreak, infectious disease, content analysis

## Abstract

**Background:**

Effective risk communication about the outbreak of a newly emerging infectious disease in the early stage is critical for managing public anxiety and promoting behavioral compliance. China has experienced the unprecedented epidemic of the coronavirus disease (COVID-19) in an era when social media has fundamentally transformed information production and consumption patterns.

**Objective:**

This study examined public engagement and government responsiveness in the communications about COVID-19 during the early epidemic stage based on an analysis of data from Sina Weibo, a major social media platform in China.

**Methods:**

Weibo data relevant to COVID-19 from December 1, 2019, to January 31, 2020, were retrieved. Engagement data (likes, comments, shares, and followers) of posts from government agency accounts were extracted to evaluate public engagement with government posts online. Content analyses were conducted for a random subset of 644 posts from personal accounts of individuals, and 273 posts from 10 relatively more active government agency accounts and the National Health Commission of China to identify major thematic contents in online discussions. Latent class analysis further explored main content patterns, and chi-square for trend examined how proportions of main content patterns changed by time within the study time frame.

**Results:**

The public response to COVID-19 seemed to follow the spread of the disease and government actions but was earlier for Weibo than the government. Online users generally had low engagement with posts relevant to COVID-19 from government agency accounts. The common content patterns identified in personal and government posts included sharing epidemic situations; general knowledge of the new disease; and policies, guidelines, and official actions. However, personal posts were more likely to show empathy to affected people (χ^2^_1_=13.3, *P*<.001), attribute blame to other individuals or government (χ^2^_1_=28.9, *P*<.001), and express worry about the epidemic (χ^2^_1_=32.1, *P*<.001), while government posts were more likely to share instrumental support (χ^2^_1_=32.5, *P*<.001) and praise people or organizations (χ^2^_1_=8.7, *P*=.003). As the epidemic evolved, sharing situation updates (for trend, χ^2^_1_=19.7, *P*<.001) and policies, guidelines, and official actions (for trend, χ^2^_1_=15.3, *P*<.001) became less frequent in personal posts but remained stable or increased significantly in government posts. Moreover, as the epidemic evolved, showing empathy and attributing blame (for trend, χ^2^_1_=25.3, *P*<.001) became more frequent in personal posts, corresponding to a slight increase in sharing instrumental support, praising, and empathizing in government posts (for trend, χ^2^_1_=9.0, *P*=.003).

**Conclusions:**

The government should closely monitor social media data to improve the timing of communications about an epidemic. As the epidemic evolves, merely sharing situation updates and policies may be insufficient to capture public interest in the messages. The government may adopt a more empathic communication style as more people are affected by the disease to address public concerns.

## Introduction

### Background

On December 31, 2019, a cluster of pneumonia cases of unknown etiology were first reported in Wuhan, the capital city of Hubei Province, China [1]. The causative pathogen was soon identified as a novel coronavirus, severe acute respiratory syndrome coronavirus 2 (SARS-CoV-2) [2] and the disease caused by SARS-CoV-2 was termed the coronavirus disease (COVID-19) [3]. Epidemiological investigation of the first 41 laboratory-confirmed human cases revealed that most had a history of visiting a seafood wholesale market (Huanan Market) in Wuhan where live wild animals were also sold for human consumption [4], but later, human-to-human transmission was confirmed, as cases without a history of visiting the market increased dramatically [5-7]. The outbreak of COVID-19 in Wuhan rapidly evolved into a severe pneumonia epidemic nationally and later worldwide. As of May 13, 2020, a total of 84,458 confirmed human cases of COVID-19 including 4644 deaths in China were reported to the World Health Organization [8]. Worldwide, COVID-19 has been a pandemic affecting more than 200 countries or territories with a total of 4,170,424 human cases of COVID-19 including 287,399 deaths as of May 13, 2020.

### Communication About Outbreaks

The outbreak of a newly emerging respiratory infectious disease usually puts individuals at a high risk of infection and constitutes a highly uncertain situation that changes rapidly, threatening serious potential loss and prompting considerable psychological distress [9,10]. Feelings of uncertainty provoke great public anxiety, which if not properly addressed can develop into public panic and herd behaviors that may harm social order and population health [11,12]. Effective outbreak communication, particularly at the early stage, becomes critically important for dealing with excessive public fear, promoting risk awareness, empowering the public in taking protective actions, and gaining public confidence and trust [11,13,14]. Conceptual models for guiding outbreak communications have been developed since the 2003 outbreak of severe acute respiratory syndrome (SARS) [14-17] and used in empirical research for examining communication practices during the 2009 influenza A/H1N1 pandemic [14], the 2013-2016 Ebola outbreak in West Africa [18], and most recently the outbreak of COVID-19 [19]. These communication models and empirical research indicate that effective outbreak communication should be prompt and transparent, dynamic as the situation evolves to meet changes in public needs, relevant and able to engage the community, and empathic and caring to address public emotional distress [14-19].

### Social Media as a Platform for Outbreak Communication

The high penetration of internet use and rapid development of information and communication technologies have made the internet an increasingly important health information source worldwide [20-23]. Reading, commenting, sharing, and seeking health information from social media, particularly through a mobile device, has become an increasingly important pattern of health information consumption in China [20,24,25]. During an epidemic, social media can facilitate the spread of epidemic awareness, attitudes toward control and preventive measures, emotional responses and behaviors, as well as misinformation and rumors in the public through online interactivity [26-30]. As the epidemic evolves, this may facilitate homogeneous mental representations of the epidemic, leading to collective behavioral responses [31]. In China, Sina Weibo (the Chinese version of Twitter) is one of the most popular platforms that attracted 486 million active monthly users in 2019 [32], most of whom accessed their user accounts through a mobile device [24]. Its microblogging function allows users to create and share short messages in a multimedia format, and other users can “share,” “like,” “comment,” and “follow” the initial posts. Numerous government agencies in China also make use of Weibo to communicate with the public. As of June 2019, there were a total of 139,270 verified government microblogs in Weibo [24].

With the proliferation of internet and social media used as health information sources, infodemiology (or infoveillance), the study (or surveillance) of “distribution and determinants of information,” in the internet or a population for the aim of guiding policy making and public health interventions [33] has been commonly used in the case of infectious disease outbreaks. Among various applications of infodemiology or infoveillance methods for social media data about infectious disease outbreaks, tracking information (concept) prevalence data [27,34-38] and qualitatively analyzing and categorizing contents of the social media data [19,27,36-41] are believed to be able to provide important insights into outbreak communications. However, existing studies mainly focused on tracking specific concepts or information such as blame [27], misinformation [34], stigma [30], specific keywords and sentiment [35], and organization trust and managing uncertainty [39], possibly due to specific research interests or using machine-aided analysis which does not allow flexible content analysis [42]. Moreover, existing studies mainly focused on one side of the outbreak communication, either the public response or response of health authorities [19,27,34-41]. This only provides partial understanding about the interactivity between the public and health authorities, whereas two-way communication is believed to be crucial for effective outbreak communication [40]. Although a recent infoveillance study on Weibo data about COVID-19 provided some qualitative descriptions about the potential interactions between the public and government online about COVID-19 by time, the study did not quantify public engagement and government responsiveness regarding COVID-19 and how these changed by time as the pandemic unfolded [38]. In addition, this study’s qualitative results seemed to lack a clear structure for understanding public perceptions and emotions [38]. The study conducted by Chew and Eysenbach [37] provides more comprehensive methods for guiding coding of social media data related to discussion topics, emotions, and online behaviors as well as quantifying and tracking the changes of these contents as the epidemic unfolds.

### Study Objectives

The outbreak of COVID-19 was an unprecedented epidemic that China experienced for the first time in this digital era. Based on this study’s literature review on important principles of effective outbreak communication and the knowledge gaps of current infodemiology or infoveillance studies examining outbreak communication using social media data, this study aimed to make use of the Weibo data about COVID-19 from December 2019 to January 2020 in China to answer the following research questions:

1. How did the public respond to the outbreak of COVID-19 as cases of COVID-19 increased and increasingly stringent containment measures were implemented, and how quickly could the government respond to public discussions about COVID-19?
2. To what extent could government messages about COVID-19 engage the general online users?
3. What contents did the public discuss online, and how did these contents change as the epidemic evolved? To what extent could the government respond to the temporal change of public discussions online?

## Methods

### Data Extraction

Four keywords in Chinese characters were used to capture data relevant to COVID-19 from Weibo from December 1, 2019, to January 31, 2020: “Wuhan pneumonia,” “novel coronavirus,” “novel coronavirus pneumonia,” and “novel pneumonia.” Data were retrieved using the built-in Weibo searching function and were subsequently screened by the Python Web Crawler, a tool that has been demonstrated to be efficient in identifying the most relevant posts that contain the set keywords [43]. A total of 1,028,204 relevant posts were initially retrieved. We also tried the keywords “unknown pneumonia” and “SARS” in Chinese characters to capture Weibo data from December 1, 2019, to January 9, 2020, when the etiology of COVID-19 was not yet confirmed [2]. Since “Wuhan pneumonia” is a less specific term for COVID-19 before official announcement of unknown pneumonia in Wuhan on January 9, 2020, we manually checked the relevant posts by January 9, 2020, in the database. This excluded 466 from 469 posts on December 30, 2019, but this term successfully detected over 99% of the posts relevant to COVID-19 in the subsequent days. The final database included a total of 1,027,738 posts comprising of 914,247 (89.0%) posts from personal accounts of the public, 45,398 (4.4%) posts from accounts of government agencies, and 67,746 (6.6%) posts from accounts of media and commercial agencies. Accounts of government, media, and commercial agencies were verified by Weibo to be “official” at registration by submitting relevant documents of their organizations for verification. Each post record comprises account name, contents, post time, engagement data of each post including numbers of likes (ie, showing confirmation or agreement with the post contents), comments, shares, and followers.

### Engagement Analysis

This study evaluated how much government posts can engage online users in the communications about COVID-19 by calculating the engagement index using engagement data of the posts delivered by government agency accounts [44,45]. The engagement data comprising likes, shares, and comments on the posts from each government account and the number of followers of these accounts were first extracted. These engagement data were then used to calculate the three metrics of social media engagement: popularity (likes per post and per 1000 followers), commitment (comments per post and per 1000 followers), and virality (shares per post and per 1000 followers); all three metrics were subsequently aggregated to generate the overall engagement index. Based on the engagement index, we identified the top five most active government agencies in both the health and nonhealth sectors. When ranking engagement, we specifically excluded any government account that delivered fewer than the average number of posts generated by all government accounts combined during the study period because these government accounts could rank high based on engagement index but were considered inactive in interacting with online audiences. We used mean but not the median number of posts as the cut-off because over 50% of government agencies only had 1 post during the study period. In addition, since the National Health Commission (NHC) of China is regarded as the lead agency in coordinating the national effort to combat the COVID-19 outbreak in China, its engagement data were included in comparison with other government agencies despite that its engagement index was not ranked in the top five.

### Content Analysis

There is currently no consensus about how to best sample social media data for content analysis [42], but random sampling has been commonly used and seems to be suitable for social media data [46]. However, for random sampling, sample sizes differ a lot across studies due to different study purposes, duration of study period, resources, and whether data were coded automatically or manually [42]. Manual coding can generate richer information by accommodating new codes emerging during data analysis, but the sample size must be kept at a manageable level to avoid fatigue and improve accuracy in coding. Since we were interested in temporal changes in the discussion contents about COVID-19 among general online users, we focused on the personal account posts for content analysis. We first excluded personal account posts that lacked all engagement (no likes, comments, and shares) because these posts may have captured little attention and interest of other online users. Thereafter, 20 posts per calendar day between December 31, 2019, and January 31, 2020, were randomly selected for content analysis. In addition, 4 posts delivered by personal accounts (1 on December 29, 2019, and 3 on December 30, 2019) before the first official announcement of the unknown pneumonia cases in Wuhan on December 31, 2019, were included for content analysis. Therefore, a total of 644 personal account posts were finally included for content analysis.

As a comparison with personal account posts and supplement for understanding government responsiveness in the communications about COVID-19, we also analyzed the contents of all posts from the first 5 government accounts of both the health and nonhealth sectors based on the rank of the engagement index and posts from the NHC in the same period. The first government post relevant to COVID-19 was posted on December 31, 2019. A total of 273 government posts were included for content analysis.

A tentative coding scheme was first developed based on preliminary analysis of a random training subset of 100 posts using open coding by one author (QL) and iteratively refined through independent analysis of another 100 training posts by authors QL, JY, and MD, and discussion from the team. The coding scheme was then used by two authors (JY and MD) to analyze the 274 selected government posts and 644 selected individual posts for final content analysis, each analyzing half of these posts. Although the coding scheme was used, the coders were advised to be open to new codes during the analysis. After both coders finished their part, they mutually checked 10% of the posts from each other’s subset to ensure consistency in coding. Finally, one author (QL) double-checked a random subset of 10% of all posts for content analysis to ensure the accuracy and reliability of the coding. Any inconsistencies were solved by going back to relevant data and joint discussions among authors QL, JY, and MD to reach an agreement. Interrater reliability was assessed by calculating Cohen kappa, with a value of 0.6 or above indicating adequate reliability. The set of codes finally generated were then constant compared to develop thematic categories.

### Statistical Analysis

A Pearson chi-square test was used to compare the overall percentages of each thematic category between personal account posts and government agency posts. Latent class analysis (LCA) was used to explore main patterns of post contents by type of account. To conduct the LCA, we first generated a binary-coded variable (1=the specific thematic category is present and 0=the specific thematic category is absent) for each thematic category coded for each post. These variables were then entered into Mplus 7.3 (Muthén and Muthén) for LCA. Major fit indices provided by Mplus including Akaike information criterion (AIC), Bayesian information criterion (BIC), sample size adjusted BIC (aBIC), and the entropy value were used to determine the optimal number of class for the post contents of both personal accounts and government agency accounts. A model with smaller values of AIC, BIC, and aBIC but with a higher entropy value is preferred, but we also consider the interpretability of the model and model parsimony [47]. The LCA would finally determine the content pattern (ie, latent class) of each post. A chi-square for trend was then used to examine the temporal change of the proportion of each content pattern by week by type of account after checking the linearity of the distribution of proportion of each content pattern by week. The temporal trend analysis excluded the 4 personal posts delivered before December 31, 2019. The LCA was conducted using Mplus 7.3, and other statistical analyses were conducted using STATA 15.1 for Windows (StataCorp LLC).

## Results

### Weibo Activity

**Figure 1:**
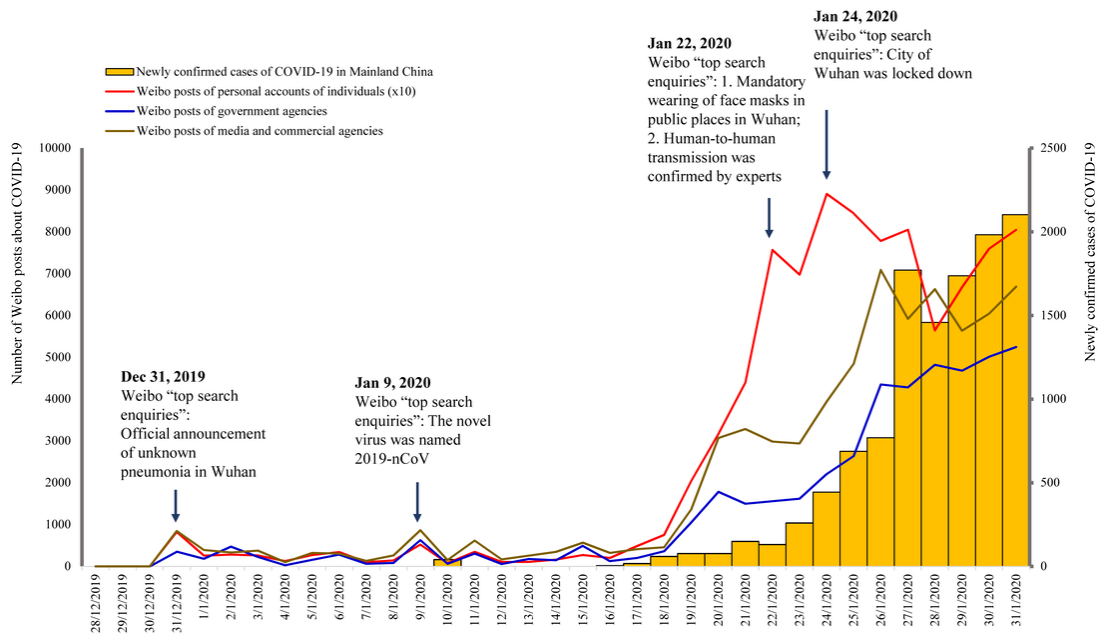
Daily numbers of newly confirmed cases of COVID-19 in Mainland China, daily numbers of Sina Weibo posts relevant to COVID-19 by account, and Weibo “top search enquiries” on peak days from December 2019 to January 2020. COVID-19: coronavirus disease; 2019-nCoV: novel coronavirus.

We detected information prevalence in relation to the outbreak by plotting daily numbers of Weibo posts by type of accounts with daily numbers of newly confirmed cases of COVID-19 in Figure 1. The Weibo “top search enquires” automatically identified by the built-in function of Weibo based on the number of relevant posts on that day were also marked on observable peak days of Weibo activity (Figure 1). There was a peak on December 31, 2019, when a cluster of unknown pneumonia cases in Wuhan were officially announced for the first time. Another small peak was detected on January 9, 2020, when there was a hot discussion about naming the etiology of unknown pneumonia in Wuhan as the novel coronavirus. Weibo activity increased dramatically starting from January 18, 2020, when daily new confirmed cases of COVID-19 substantially increased. For personal account posts, a third peak was found on January 22, 2020, when the Wuhan government announced a policy of mandatory wearing of face masks in public places and human-to-human transmission of COVID-19 was announced by an expert who had just visited Wuhan for investigations, and the last peak within our study timeframe was detected on January 24, 2020, within 24 hours after Wuhan city was locked down. For posts from government agencies and media and commercial agencies, the third peak was not observed, and the last peak within the study time frame occurred 2 days later. Overall, it appeared that public online reactions followed the spread of the disease and government actions, and responded earlier than the government.

### Public Engagement With Government Messages

Table 1 presents the engagement metrics of the five most active Weibo accounts of government agencies from both the health and nonhealth sectors in the communications about COVID-19 on Weibo. In the health sector, the most active government agencies are the Municipal Health Commission (MHC) in several cities of China, including Wuhan, Zhuhai, Shanghai, and Beijing, and one city-level hospital in Sichuan Province, China. In China, the MHC in different cities are required to report duties to the provincial and national health commissions. In the nonhealth sector, the most active government agency was the Hubei Branch of the Red Cross Society of China, an organization supervised by the Chinese government that is mainly responsible for encouraging donation to support affected people during the epidemic. The remainders are from the system of public security bureaus that are responsible for tracking close contacts of patients with COVID-19 and implementing local policies of traffic restrictions during the epidemic. The engagement analysis shows that, despite being relatively more active compared with other government agency accounts based on the rank of the engagement index, other than Wuhan MHC, all government agencies had low popularity (likes per post per 1000 followers), commitment (comments per post per 1000 followers), and virality (shares per post per 1000 followers). The NHC, despite having attracted a large number of followers, had extremely low popularity, commitment, and virality.

**Table 1:** Government agencies with the greatest engagement and associated engagement metrices for coronavirus disease communications from December 2019 to January 2020.

Government agencies		Posts, n		Followers, n		Popularity^a^, n		Commitment^b^, n		Virality^c^, n		Engagement index^d^	
**Top 5 from health sector**													
	Wuhan MHC^e^		40		58,144		242.73		9.72		2.62		255.07
	Zigong No 4 People’s Hospital		21		298		42.03		5.27		0.96		48.26
	Zhuhai MHC		18		11,844		27.82		3.53		1.18		32.52
	Shanghai MHC		11		403,603		25.35		1.84		1.14		28.34
	Daxing (in Beijing) MHC		12		65,707		14.41		1.61		1.38		17.41
**Top 5 from nonhealth sector**													
	Hubei Branch of the Red Cross Society of China		21		97,523		78.20		8.70		1.10		88.01
	Gaolan People’s Procuratorate (in Gansu Province)		22		550		13.06		4.71		1.82		19.59
	Suixian People’s Procuratorate (in Hubei Province)		59		435		6.97		4.71		2.42		14.10
	Longchang Public Security Bureau (in Sichuan Province)		26		8803		12.51		0.73		0.20		13.44
	Datong Fire Services Department (in Shanxi Province)		13		2155		8.07		2.75		0.82		11.64
National Health Commission of China		30		5,371,595		7.51		0.33		0.22		8.04	

^a^Likes per post per 1000 followers.

^b^Comments per post per 1000 followers.

^c^Shares per post per 1000 followers.

^d^Engagement index = popularity + commitment + virality.

^e^MHC: Municipal Health Commission.

### Individual and Government Post Contents

Frequencies and proportions of thematic categories of post contents for personal and government accounts are shown in Table 2, and detailed descriptions of the thematic categories identified in our study can be found in Multimedia Appendix 1.

We noted 3 cyber-support behaviors from personal account posts: *sharing knowledge or information*, *emotional exchange,* and *seeking information* of which only the first 2 were identified in government posts. As is shown in Table 2, for *sharing knowledge and information* in both groups, the most common thematic categories were situation updates of COVID-19 followed by general knowledge about coronavirus pneumonia and advice on preventive measures. Government agency posts were more likely to share information about policies, guidelines, and official actions (χ^2^_1_=14.5, *P*<.001), and instrumental support (χ^2^_1_=32.5, *P*<.001), while personal account posts were more likely to share information on public responses to the epidemic (χ^2^_1_=19.1, *P*<.001). Personal account posts were more likely to be classified as *emotional exchange* (χ^2^_1_=30.5, *P*<.001) including showing empathy to affected people (χ^2^_1_=13.3, *P*<.001), attributing blame to people or organizations for malpractice during the epidemic (χ^2^_1_=28.9, *P*<.001), and expressing worry about the epidemic (χ^2^_1_=32.1, *P*<.001). The government posts more likely praised people or organizations (χ^2^_1_=8.7, *P*=.003). The main groups of people praised by both groups were health care workers, while the main people or organizations being blamed in personal account posts included other individuals (eg, individuals who consumed wild animals, breached the infection containment measures, and committed medical violence) and the government (individual government officers or government in general). Regarding *seeking information*, the main information sought was updates about the epidemic situation.

**Table 2:** Frequency of thematic categories from posts delivered by individual and government accounts.

Thematic categories		Individuals (n=644), n (%)	Government (n=273), n (%)	*P* value^a^
**Sharing knowledge/information**		567 (88.0)	258 (94.5)	.003
	Situation update about COVID-19^b^	287 (44.6)	108 (39.6)	.16
	General knowledge about coronavirus pneumonia	206 (32.0)	82 (30.0)	.39
	Advice on preventive measures	114 (17.7)	56 (20.5)	.32
	Policies, guidelines, and official actions	95 (14.8)	69 (25.3)	<.001
	Human-to-human transmission	79 (12.3)	27 (9.9)	.30
	Fight against rumors	46 (7.1)	23 (8.4)	.50
	Cause of viral emergence	44 (6.8)	14 (5.1)	.33
	Public response during the epidemic	43 (6.7)	0 (0.0)	<.001
	Instrumental support	13 (2.0)	29 (10.6)	<.001
	Infection and illness experience	10 (1.6)	2 (0.7)	—^c^
	Seeking social support	10 (1.6)	0 (0.0)	—^c^
	Request for information transparency	8 (1.2)	0 (0.0)	—^c^
	Reports of scientific research	3 (0.5)	0 (0.0)	—^c^
	Seeking close contact	0 (0.0)	2 (0.7)	—^c^
**Emotional exchange**		321 (49.8)	82 (30.0)	<.001
	Showing empathy to or blessing affected people	86 (13.4)	14 (5.1)	<.001
	Blaming people or organizations	78 (12.1)	3 (1.1)	<.001
	Providing reassurance about risk	73 (11.3)	30 (11.0)	.88
	Expressing worry or fear about the risk	70 (10.9)	0 (0.0)	<.001
	Praising people or organizations	53 (8.2)	40 (14.7)	.003
	Warning about the epidemic	48 (7.5)	11 (4.0)	.05
Seeking information		36 (5.6)	0 (0.0)	<.001

^a^*P* values were calculated using a Pearson chi-square test.

^b^COVID-19: coronavirus disease.

^c^Cell with expected frequency less than 5 and thereby *P* values from the chi-square test were not available.

Compared with post contents of government agencies from the health sector, we found that the four government agencies from the system of public security bureaus were more likely to share information about situation updates (χ^2^_2_=15.9, *P*<.001), while the Hubei Red Cross Society of China were more likely to post contents about instrumental support (eg, donation of materials or money; χ^2^_2_=25.3, *P*<.001) and showing empathy to affected people (χ^2^_1_=25.7, *P*<.001).

### Content Patterns and Temporal Changes

The LCA revealed five main content patterns within both personal posts and government posts (Multimedia Appendix 2). For personal posts excluding the 4 posts before December 31, 2019, the most prevalent content pattern comprised of posts that were mainly sharing situation updates (situation class; n=208/640, 32.5%), followed by those with greater probabilities of showing empathy and blaming (empathy-blaming class; n=166/640, 25.9%); those mainly sharing general knowledge (knowledge class; n=119/640, 18.6%); those mainly sharing policy, guidelines, and official actions (policy class; n=85/640, 13.3%); and those mainly sharing worry about the epidemic (worry class; n=62/640, 9.7%). For the government posts, 3 similar classes as those in the personal posts were found, including the situation class (n=77/273, 28.2%), knowledge class (n=79/273, 28.9%), and policy class (n=40/273, 14.7%; Multimedia Appendix 3). Another 3 classes were different from those of the personal posts, including posts that mainly shared prevention tips and fought against rumors (prevention-rumors class; n=40/273, 14.7%) and those mainly providing instrumental support, praising people, and that had a slightly higher probability of showing empathy (support class; n=37/273, 13.6%; Multimedia Appendix 3).

**Figure 2:**
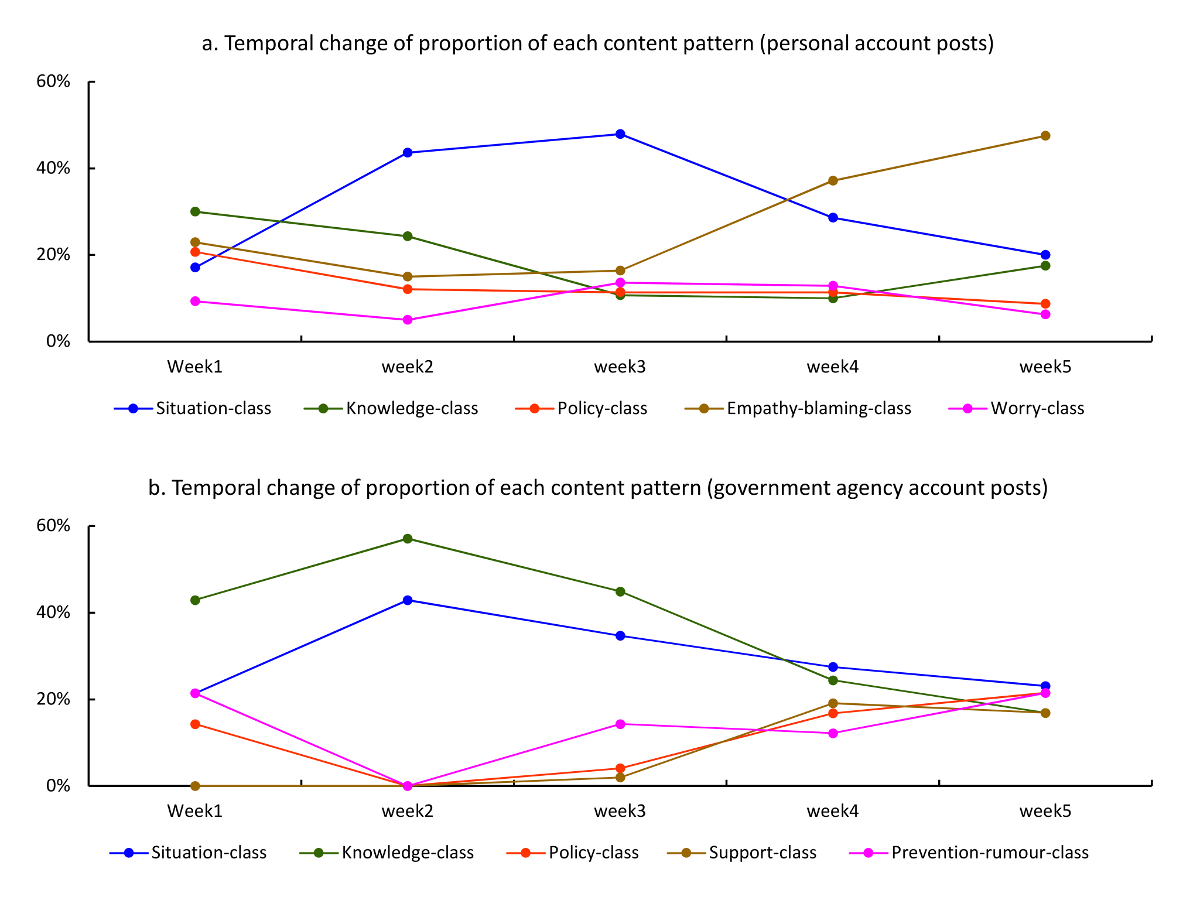
Temporal changes in the proportion of main content patterns by type of account.

The temporal change of each content pattern by type of account are shown in Figure 2. The chi-square for trend analyses found that for personal posts, proportions of posts sharing situation updates increased from week 1 (December 31, 2019-January 6, 2020) to week 3 (January 14-20, 2020; for trend, χ^2^_1_=28.6, *P*<.001) but declined thereafter (for trend, χ^2^_1_=19.7, *P*<.001). Sharing general knowledge (for trend, χ^2^_1_=15.3, *P*<.001) and policies, guidelines, and official actions (for trend, χ^2^_1_=15.3, *P*<.001) declined each week, while showing empathy and blaming increased significantly in later weeks (for trend, χ^2^_1_=25.3, *P*<.001), and worry about the epidemic remained stable and low. For government agency account posts, sharing situation updates and prevention tips, and fighting against rumors remained stable; sharing general knowledge declined each week (for trend, χ^2^_1_=14.7, *P*<.001), while sharing policies, guidelines, and official actions (for trend, χ^2^_1_=8.9, *P*=.003), and providing instrumental and emotional empathy and praising people (for trend, χ^2^_1_=9.0, *P*=.003) increased significantly on the last 2 weeks of the study time frame.

We also specifically explored how providing reassurance, a main emotional content in government posts, changed as the epidemic evolved. We found that providing reassurance in government posts was more frequent in the first 3 weeks but declined in the last 2 weeks of the study time frame (for trend, χ^2^_1_=4.2, *P*=.04)

## Discussion

### Principal Findings

The Weibo activity in the early stage of the COVID-19 epidemic showed that public reactions seemed to follow the spread of the disease and government actions in China. This again evidences the value of infodemiology or infoveillance studies to understand public response to the disease and government actions during the early epidemic stage despite a concern over the censorship of online information for propaganda purposes in China. There was 1 post blaming people who consumed wild animals for causing “Wuhan pneumonia” that was captured on December 29, 2019. On December 30, 2019, 3 more posts were identified: 2 seeking confirmation about unknown pneumonia cases detected in Hunan Seafood Market in Wuhan and 1 sharing information about unknown pneumonia cases found in the market. All 4 posts were from accounts of individuals who lived in Wuhan. This indicates that before the official announcement of the first cluster of unknown pneumonia in Wuhan on December 31, 2019 [1], relevant information had been spread in the public through interpersonal communication, social media, or other channels. The first government post was delivered on December 31, 2019, 2 days after the first individual post, indicating that, although the Chinese government’s outbreak communication has been more timely and transparent compared with their response to the 2003 SARS outbreak [48], more efforts are required to improve early outbreak communication when uncertainty usually challenges communication. Early posts should be treated as alarms and responded to in a timely manner rather than being silenced, which could receive harsh criticism and worsen the epidemic control [49].

Our study evidences the use of social media among the Chinese government agencies in the communications about COVID-19 at the early epidemic stage, but the communication remained limited. The engagement analysis indicates that the public generally had low engagement with government agency posts, even those from the most active government agencies. In China, the NHC is expected to play a leading role in risk communication during an epidemic, while provincial and municipal health commissions are expected to report duties and provide epidemic information of their own provinces and cities, respectively, to the NHC. However, although the NHC and provincial health commissions have attracted a large number of followers on social media during the epidemic, compared with municipal health commissions, messages from these high-level health authorities were more disengaged by the general public. The generally low values of the engagement metrics popularity (likes per post per 1000 followers), commitments (comments per post per 1000 followers), and virality (shares per post per 1000 followers) may indicate low levels of interest, use, emotional arousal, or even credibility of the government information among the online audiences [50,51]. This may partly reflect a population inertia effect, where it takes a certain amount of time, or threat, before there is a noticeable change in the bulk practices of a population. The duration of such a period of inertia would be valuable to know.

The content analysis suggests that the Chinese government agencies mainly used social media to “inform” the public about updates of the epidemic situation; knowledge of the coronavirus pneumonia; policies, guidelines, and government actions; and prevention tips, all being the key risk messages included in the official websites of health authorities for communicating about an epidemic [40]. This suggests that government agencies mainly adopt a top-down approach in risk communication and use the social media for one-way communication. The temporal changes of content patterns of personal posts indicate that the public have less interest in situation updates, general knowledge, policies, and guidelines as the epidemic evolves. However, the public may feel more empathic with the affected people and angry about other individuals or the government who put people at risk, as an increasing number of people are affected by the disease and the control measures. The government seemed to continue frequently sharing situation updates; policies, guidelines, and official actions; and prevention tips despite a decline in public interest. However, we also observed a significant increase in the frequency of instrumental and emotional support in government posts as the epidemic evolved. This provides in-depth understanding about why sentiment analysis indicates a decline in negative sentiment but increases in positive sentiment as the epidemic unfolded [35]. The inconsistent temporal changes in content patterns between personal and government posts and insufficient emotional support of government posts indicates an overall inadequate government responsiveness to public concerns. Reassuring the public about the epidemic risk was one of the main emotional contents identified from the government posts and was particularly apparent in the first 2 weeks; even Weibo data indicated a generally low risk awareness and concern among the general public. This is consistent with the public response to the 2009 influenza A/H1N1 pandemic shown by Twitter data [37]. Our study indicates that this may be because the government overreassured the public at the early epidemic stage, which is against Sandman and Lanard’s [52] guidelines that risk communication should lean toward the alarming side particularly when the situation is uncertain. However, reassurance should be provided as more people are infected to deal with excessive or prolonged fear. The increasing use of blame in personal posts as more people were infected coincides with Douglas’ [53] idea that contemporary risk is highly politicized. As more people are affected, “whose fault?” becomes a primary question to seek the accountability of certain persons or organizations and make sense of the epidemic [27]. Our study found that the public blamed not only individuals who put others at greater risk during the epidemic but also government, particularly local government figures for their perceived failures in risk communication and control measures. This also aligns with Beck’s works on global risk society, in which authorities are increasingly questioned and blamed for failing to protect individuals [54]. Current data reveals seldom use of conspiracy theories in the attribution of blame and blame as a way to spread rumors [55]. In contrast, the government agencies tend to praise people who had made contributions to the control of the epidemic. This is viewed as a blame-avoidance strategy, called heroization [55], to direct public outrage to the appreciation of another group of people. Working with heroes in outbreak communication may be critical to improving communication effectiveness.

### Limitations

First, individual posts were sampled by randomly choosing equal numbers of posts per calendar day for content analysis rather than using the common probability-based random sampling, which would generate a large sample size due to the vast amount of social media data. However, there is currently no consensus about how to best sample social media data, and our sampling strategy was able to draw a random manageable sample using manual coding. Second, only posts of government agencies that were more actively engaged with the public in online communication about COVID-19 were included for content analysis. This means that the sample of government posts for content analysis may not be representative of all government posts. However, this sample was considered to have greater impact on online audience’s knowledge, perceptions, attitudes, and behaviors due to greater audience interest and attention to their messages. Third, our study did not evaluate the responses to and concerns over COVID-19 among nonnetizens, particularly those living in rural areas and older people in China [24], and the Chinese government’s communications about COVID-19 through other channels. In addition, our data only covered the first 5 weeks after unknown pneumonia cases in Wuhan were first officially announced, which is a relatively short but critical period for outbreak communication. Furthermore, due to the problem of censorship on Weibo data in China, our data based on keyword extraction may lose a considerate part of Weibo data, particularly those posted before the official announcement of the outbreak in Wuhan. This means that our data may not be able to accurately assess the delay of government responsiveness to the threat online.

### Implications

The governments can closely monitor the social media discussions to identify public concerns to further improve government responsiveness in outbreak communication. To improve government responsiveness and public engagement, first, persons who have received training in risk communication can be designated for monitoring public concerns online, delivering timely messages, communicating about the uncertainty, and making efforts to address public concerns online. Second, the municipal health commission can communicate more locally relevant information to attract local followers’ interest and motivate their information sharing. Third, the provincial and national health commissions can organize direct dialogues with online audiences on social media (eg, Weibo Chats) with trustworthy health care workers to capture audience’s attentions and interests, and facilitate the rapid spread of fact-related messages [39]. Although the main role of the NHC in risk communication may be to disseminate facts, increasing messages showing empathy and care to affected people as the epidemic evolves is believed to be essential for maintaining credibility and trust in the public during a crisis [14].

### Conclusion

The public seemed to respond earlier to the outbreak of COVID-19 online than government agencies. TThe Chinese government agencies’ use of social media for outbreak communications remained limited to providing knowledge and information to the public. As the epidemic evolved, the public had declining interest in fact-related messages but became more empathic with the affected people and tended to attribute blame to other individuals or the government. The tendency of increasingly attributing blame to other individuals or the government may push the Chinese government to seek accountability and refine the compensation system for affected people. As more people are affected, the government may adopt a more empathic communication style to address public emotional response.

## Appendix

Multimedia Appendix 1 Descriptions of thematic categories of post contents.

Multimedia Appendix 2 Comparing model fit indices of latent class analysis models with different numbers of latent class by personal accounts and government agency accounts.

Multimedia Appendix 3 Item-response probabilities of each content pattern of the five-class model by personal accounts and government agency accounts.

## Abbreviations

aBIC: adjusted Bayesian information criterion
AIC: Akaike information criterion
BIC: Bayesian information criterion
COVID-19: coronavirus disease
LCA: latent class analysis
MHC: Municipal Health Commission
NHC: National Health Commission
SARS: severe acute respiratory syndrome
SARS-CoV-2: severe acute respiratory syndrome coronavirus 2
