# Supplementary material for: Public Engagement and Government Responsiveness in the Communications About COVID-19 During the Early Epidemic Stage in China: Infodemiology Study on Social Media Data
Source: J Med Internet Res. 2020 May 26;22(5):e18796. doi: 10.2196/18796 (PMC7284407; doi:10.2196/18796)
Supplement: Multimedia Appendix 1 [file jmir_v22i5e18796_app1.docx]

Multimedia Appendix 1 Descriptions of thematic categories of post contents

| Thematic category | Descriptions |
| --- | --- |
| Situation of COVID-19 update | Post or repost official updates about the latest epidemic situation of COVID-19 mostly about number of newly confirmed cases or death cases as well as some epidemiological backgrounds. |
| General knowledge about coronavirus pneumonia | Posts sharing knowledge about coronavirus, pneumonia, or coronavirus pneumonia including their aetiologies, clinical symptoms, clinical consequences, general treatment and patient management and history of viral evolvement. |
| Advice on preventive measures | Posts providing advices on individual preventive measures during the epidemic. |
| Policies, guidelines and official actions | Posts sharing information about policies, guidelines  or official actions in response to COVID-19 in China or other countries. This also include the policies, guidelines and official actions suggested by international organizations such as UN and WHO (e.g. travel restrictions). |
| Human-to-human transmission | Posts mentioning whether the novel virus can be transmitted between humans |
| Fight against rumours | Posts that clarify the rumours or warn about any harmful impacts of spreading rumours. |
| Cause of viral emergence | Posts that sharing information about the source of the novel virus (e.g. wild animals) or behaviours (e.g. consumption of wild animals), environmental factors (e.g. climate change) or political purposes (e.g. conspiracy explanations) that cause the emergence of the virus. |
| Public response during the epidemic | Posts sharing information about the general public’s behavioural reactions to the epidemic or description of their daily activities during the epidemic. |
| Instrumental support | Posts sharing information about monetary, informational, material or man power supports for the affected people during the epidemic. |
| Infection and illness experience | Posts that share stories about the illness or infection experience of individual patients and their family members. This is distinguished from official reports about the epidemiological backgrounds of the patients. |
| Request for information transparency | Posts that demand for timely and more transparent information from authorities. This is distinguished from seeking information because these posts were not aimed for seeking specific information. |
| Reports of scientific research | Posts sharing scientific reports about the COVID-19. |
| Showing empathy to or blessing affected people | Posts expressing empathy to affected people, providing encouragement or good wishes to the general public or people in Wuhan. |
| Blaming people or organizations | Posts attributing blame to other individuals (e.g. individuals who consumed wild animals, breached the infection containment measures and committed medical violence) or the government (individual government officers or government in general) during the epidemic. |
| Providing reassurance about risk | Posts expressing confidence in the control of the epidemic or that the novel disease is not severe, or ask others not to worry/fear about the epidemic. |
| Expressing worry or fear about the risk | Posts expressing worry or fear about the risks of COVID-19. |
| Praising people or organizations | Posts expressing appreciation or compliment to healthcare workers, government, industrial employers, others individuals or media for their contribution in combatting the COVID-19 epidemic. |
| Warning about the risk | Posts warning about the severity of COVID-19, easy transmission of the infection or asking people to raise their risk awareness. |
| Seeking information | Posts asking specific information about the COVID-19 or the epidemic. |
