# Supplementary material for: Public Engagement and Government Responsiveness in the Communications About COVID-19 During the Early Epidemic Stage in China: Infodemiology Study on Social Media Data
Source: J Med Internet Res. 2020 May 26;22(5):e18796. doi: 10.2196/18796 (PMC7284407; doi:10.2196/18796)
Supplement: Multimedia Appendix 2 [file jmir_v22i5e18796_app2.docx]

Multimedia Appendix 2: Comparing model fit indices of LCA Models with different number of latent class by personal accounts and government agency account

| LCA model with different number of classes by type of account | AIC | BIC | aBIC | Entropy |
| --- | --- | --- | --- | --- |
| Personal account posts |  |  |  |  |
| 1-class model | 6187.80 | 6243.80 | 6204.53 | - |
| 2-class model | 6123.09 | 6243.55 | 6157.83 | 0.77 |
| 3-class model | 6096.76 | 6279.68 | 6149.51 | 0.73 |
| 4-class model | 6069.74 | 6314.81 | 6140.19 | 0.86 |
| 5-class model ^a^ | 6043.74 | 6351.58 | 6132.51 | 0.89 |
| 6-class model | 6029.88 | 6400.18 | 6136.66 | 0.83 |
| Government agency account posts |  |  |  |  |
| 1-class model | 2267.75 | 2303.84 | 2272.14 | - |
| 2-class model | 2185.19 | 2260.99 | 2194.40 | 0.78 |
| 3-class model | 2152.31 | 2267.82 | 2166.35 | 0.76 |
| 4-class model | 2133.44 | 2288.65 | 2152.31 | 0.94 |
| 5-class model ^b^ | 2108.99 | 2303.90 | 2132.68 | 0.97 |
| 6-class model | 2102.94 | 2337.55 | 2131.45 | 0.99 |
| 7-class model | 2109.95 | 2384.27 | 2143.30 | 0.97 |

AIC: Akaike information criterion; BIC: Bayesian information criterion; aBIC: Sample size adjusted BIC. Entropy: Entropy value which ranges from 0 to 1.

^a^ This model with the lowest value of aBIC and highest entropy value. As the 6-class model was tried, values of both BIC and aBIC increased and entropy value declined.

^b^ Although the 6-class model with the lowest value of aBIC and highest entropy value, the 5-class model was more interpretable and more parsimonious and thereby was chosen.
