# Supplementary figures and images for: Public Engagement and Government Responsiveness in the Communications About COVID-19 During the Early Epidemic Stage in China: Infodemiology Study on Social Media Data
Source: J Med Internet Res. 2020 May 26;22(5):e18796. doi: 10.2196/18796 (PMC7284407; doi:10.2196/18796)

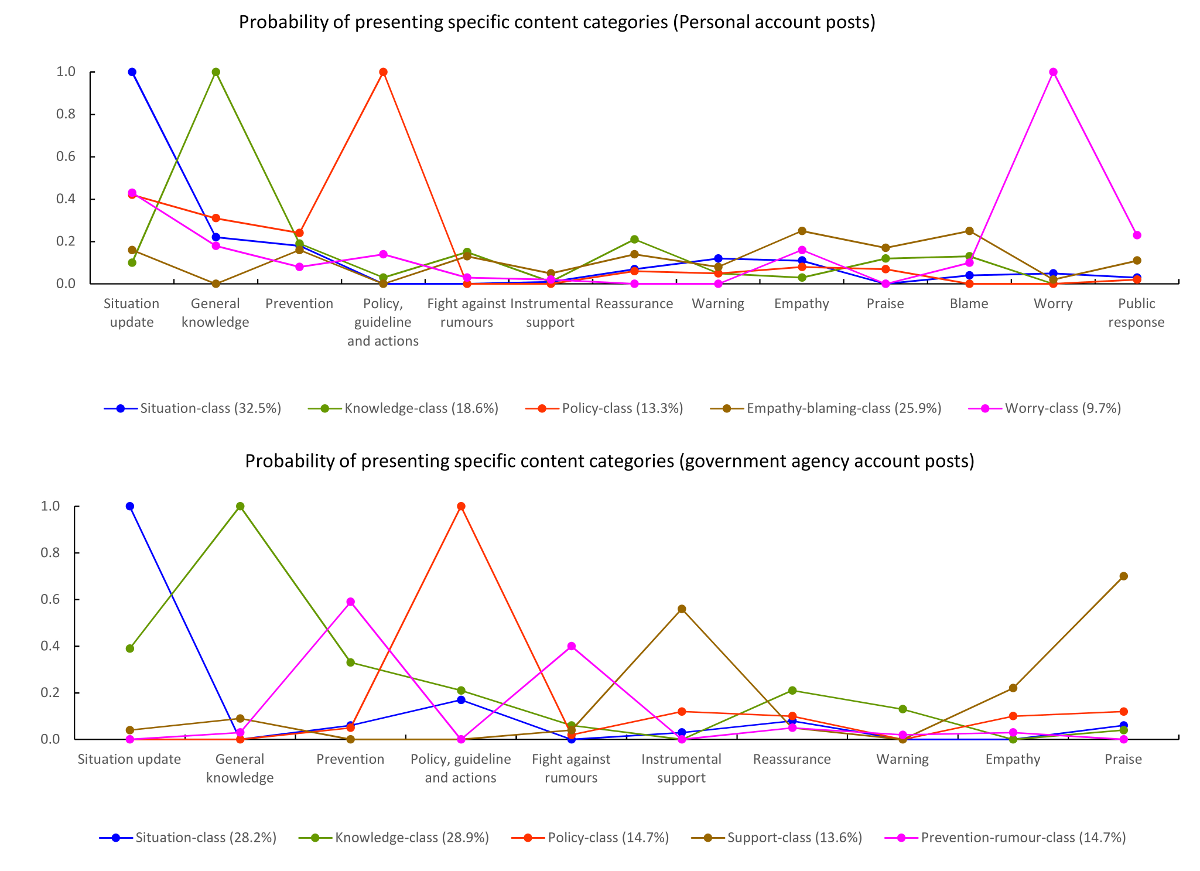

Supplement: Multimedia Appendix 3 [file jmir_v22i5e18796_app3.png]
